# The Emerging Role of Artificial Intelligence in Heart Failure

**DOI:** 10.1080/14796678.2025.2523155

**Published:** 2025-07-03

**Authors:** Brett S Bernstein, Sona Streather, Kevin O’Gallagher

**Affiliations:** 1 School of Cardiovascular and Metabolic Medicine & Sciences, British Heart Foundation Centre of Research Excellence, King’s College London, London, UK; 2 King’s College Hospital NHS Foundation Trust, London, UK; 3 Bart’s Health NHS Trust, London, UK

**Keywords:** Heart failure, Biomarkers, Echocardiography, Imaging, Pharmacogenetics

## Abstract

Heart Failure is a prevalent disease with significant impacts on morbidity and mortality. Heart failure patients have a large volume of healthcare data which is digitised and can be collated. Artificial intelligence (AI) can then be used to assess the data for underlying patterns.

AI systems can be trained to analyse readily available data, such as ECGs and heart sounds, and assess likelihood of heart failure. AI can also risk stratify heart failure patients by analysing available healthcare data.

AI can allow rapid assignment of heart failure patients to specific groups via automated echo analysis, but can also provide information regarding novel imaging bio-markers that may be more useful than left ventricular ejection fraction, such as first phase ejection fraction.

AI can be used to assess patients’ suitability for existing drugs, whilst also enabling development of novel drugs for known or newly discovered drug targets.

Heart Failure as a field, with its multi-modal data set and variability in outcomes, will greatly benefit from the expansion and improvement of AI technology over the next twenty years.

## 1 Introduction

Artificial Intelligence (AI) is a broad term that describes the novel ability of computer-based systems to mimic human cognitive function, and thereby solve complex problems previously thought to be solvable only by human intelligence[1]. Since the mid 1900s, ever-more sophisticated computers have been able to handle larger amounts of data, find patterns, and begin to learn.

Modern day medicine produces a vast amount of data on an individual and population level. For example, each individual will have observations, blood results, ECGs, scans, a medical history including medications, and demographic data. The digitisation of healthcare provision and its record keeping allows the formation of large scale data sharing networks, which can then be utilised by healthcare services and society at large for further analysis.

Understanding patterns and trends within this vast array of data can be challenging for a variety of reasons, including the large numbers of variables and subjects in the data set, and the complexity or subtlety of the trends in question. Different forms of AI lend themselves to varied data sets. For example, Natural Language Processing enables machines to interpret words from voice or text, whereas Convolutional Neural Networks are more geared towards image analysis. A brief overview of AI terms can be found in Table 1.

Heart Failure (HF) is a perfect example of a disease which has already, and will continue to benefit from AI input. It has a high worldwide prevalence, with a tendency towards multi-modal data. There are multiple evidence-based treatments, but assessing which patients will benefit from which medical interventions can be difficult to establish. Our guidelines often rely on fixed arbitrary cut-offs which can overlook the nuance of individual cases, and make outcome prediction difficult. One example is that guidelines suggest that heart failure patients with a medically optimised left ventricular ejection fraction of <35% should receive a primary prevention implantable cardioverter defibrillator (ICD)[2] but the majority of patients who receive an ICD for primary prevention never receive any device therapy[3].

**Figure 1:**
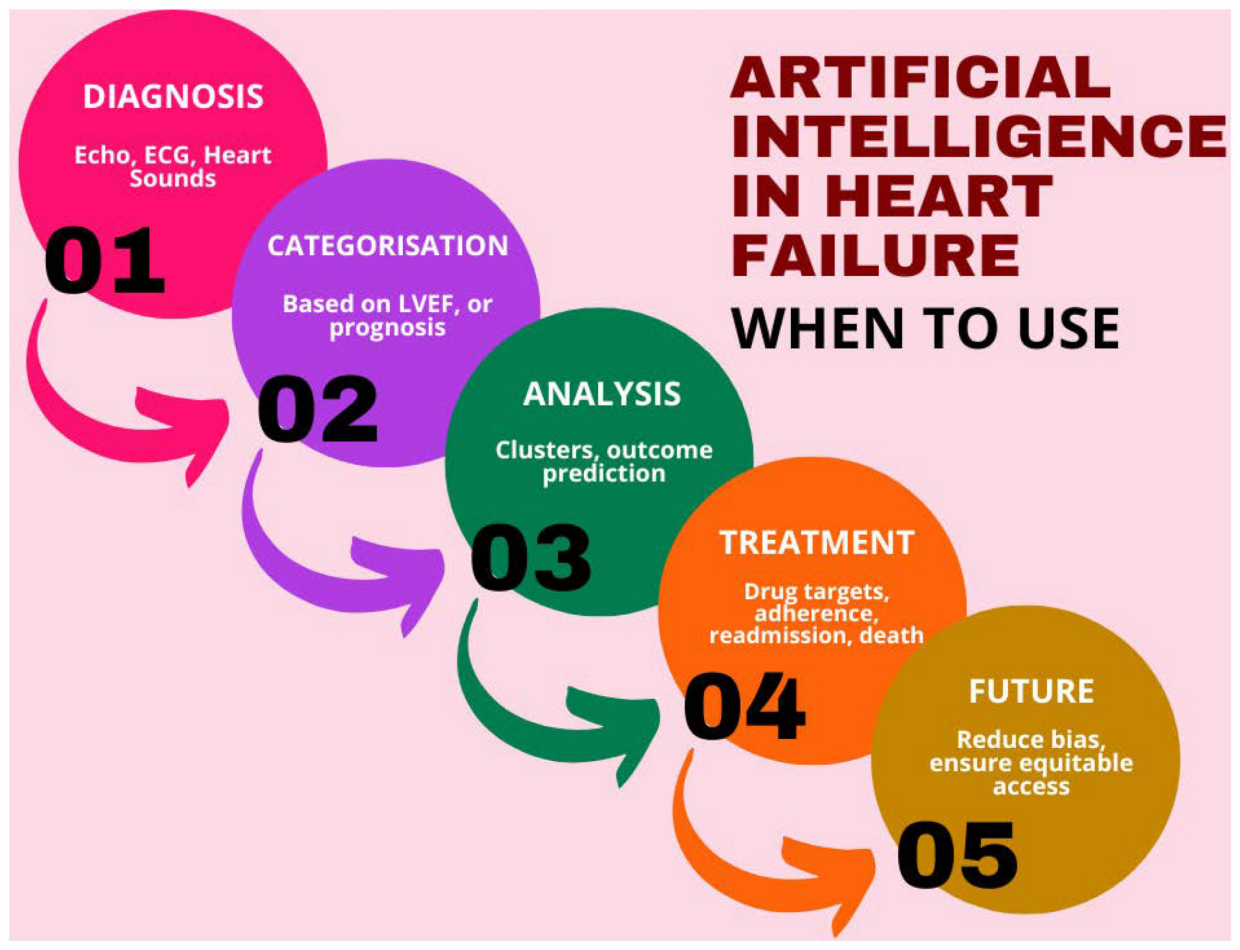
Flow chart of ArtificiaI Intelligence uses in Heart Failure. Echo – Echocardiogram ECG – Electrocardiogram LVEF – Left ventricular ejection fraction

This review will discuss the following roles for AI in HF (See Figure 1):

1) Diagnosis
  a) Diagnosing HF from simple, cheap data (eg ECG, heart sounds)
  b) Risk stratifying from available healthcare data
2) Categorisation
  a) Using AI to categorise HF patients into existing groups such as reduced ejection fraction v preserved ejection fraction
  b) Using AI to develop new HF categories or phenogroups, using existing or novel biomarkers, which may more accurately stratify risk or indicate suitability for specific treatments
  c) Using AI to analyse electronic hospital records and assessing likelihood of HF
3) Treatment
  a) Assessing patients’ suitability for existing drugs
  b) Discovering new drugs for existing drug targets
  c) Discovering new drug targets

## 2 Diagnosis

Electrocardiograms (ECGs) have been one of the most important tools in cardiovascular medicine for many years. They provide a fast, non-invasive and widely available test used in most healthcare settings worldwide. The ECG is used to diagnose ischaemia, arrhythmias and structural abnormalities and provides clues to systemic illnesses like electrolyte abnormalities. Human interpretation of ECGs varies widely depending on skill and experience as well as factors such as time constraints and a busy, distracting environment.

Artificial intelligence (AI) methods, specifically deep-learning convolutional neural networks (CNN), have been adapted to analyse the 12-lead ECG[4]. In addition to enabling automation of ECG analysis, AI-enhanced ECGs have been able to detect ECG patterns that humans cannot see. These patterns can reveal heart disease including hypertrophic cardiomyopathy and left ventricular systolic dysfunction (LSVD) in asymptomatic patients[5]. Machine learning has also been used to predict heart failure from heart rate variability data, using methods such as decision trees and support vector machines[6, 7].

Kwon et al[8] developed a deep-learning algorithm for heart failure detection from ECGs, using a deep neural network with 5 layers, as they found no gain in accuracy with more than 5 layers. A rectified linear unit was used as the activation function[8]. This was compared to conventional machine-learning based algorithms (linear regression and random forest), and outperformed both of these with respect to both sensitivity and specificity.

Earlier diagnosis of LVSD will lead to earlier commencement and optimisation of medical therapy and therefore reduced morbidity and mortality from heart failure[4].

Adidensewo et al[9] undertook a randomised clinical trial to assess the impact of using AI-enhanced digital stethoscopes to identify LVSD in pregnant and post-partum women. Identification of cardiomyopathy in this group was confirmed using echocardiography. They concluded using AI-enhanced stethoscopes improved diagnosis of pregnancy-related cardiomyopathy. Jiang et al.[10] have also trained a deep neural network to effectively diagnose valvular heart disease, achieving a sensitivity of 93% and specificity of 81%. This is particularly prescient in light of declining auscultation skills among medical professionals[11], well-documented low sensitivity and specificity of precordial auscultation in valvular heart disease detection[12, 13], and concerns regarding accurate documentation of auscultation findings.

Echocardiography is another cornerstone of the diagnostic toolbox used in cardiology, and again widely available, but conventionally requires specialist training to operate machinery and interpret images. Interpretation of images varies depending on operator skill and patient anatomy[1]. AI is already integrated into some echo equipment and has impacts on efficiency and more accurate diagnoses[14].

Machine learning (ML) has generated a training algorithm to estimate LVEF by using a database of more than 50000 echo studies. The results of this showed a similar accuracy in detecting EF when compared with skilled clinicians[15].

Narang et al[16] used a deep-learning (DL) algorithm that enabled novices in echocardiography to acquire images using step-by-step instructions from the software. The resulting images were deemed to be of good diagnostic quality, similar to those images acquired by trained clinicians scanning the same patients. These advances have important implications in allowing diagnostic tools to be used in resource poor settings with fewer specialised clinicians.

Deep learning models can be trained on echocardiography images and data to measure patterns predictive of heart failure and identify subtle changes that may be invisible to the human eye[17] which may allow for earlier detection of heart failure or conditions that lead to heart failure. This has benefits for risk stratification and earlier interventions to prevent or delay the development of heart failure.

These AI models can be used in both traditional echo machines and handheld devices. When used with handheld devices this could allow for rapid diagnosis and prediction of predilection to heart failure, which could have a meaningful impact on expediting drug therapies that may affect cardiac function, such as some chemotherapy drugs.

Using AI-enabled echocardiography devices may have a meaningful impact on cost savings in terms of accurately interpreting the same information using lower cost machines and less-skilled technicians. There is potential that this cost saving could lead to a wider availability of echocardiography in resource-poor settings.

The global registry of acute coronary events (GRACE) scoring system has been used for around twenty years to risk-stratify patients with acute coronary syndrome (ACS) in order to guide individualised patient management. The GRACE score 2.0 was found to underestimate the risk of in-hospital mortality in female patients. The redeveloped GRACE score, GRACE 3.0 has incorporated machine learning to incorporate sex differences and risk-stratify ACS patients more effectively[18, 19].

Machine learning has been used to create risk scores to predict mortality from heart failure in patients using data available in electronic health records (EHRs). Variables include blood markers such as creatinine and physiological variables like diastolic blood pressure[20].

Using AI models for both ECG and echo interpretation can be combined with AI information from patients’ electronic healthcare records, such as age, sex, and medical history to further risk-stratify their risk of developing heart failure.

## 3 Categorisation

Categorisation and classification of patients into specific groups, and syndromes into specific diagnoses is a cornerstone of modern medicine.

There are many ways of categorising heart failure. Most commonly, left ventricular ejection fraction is used to delineate these: HF with preserved, moderately reduced, and reduced ejection fractions respectively (HFpEF, HFmrEF, & HFrEF). Indeed, as discussed above, with AI-derived automated echocardiographic reporting software, this distinction can be made rapidly.

It has been demonstrated that left ventricular ejection fraction (LVEF) does not necessarily correlate with left ventricular contractility[21]. However, a left ventricular ejection fraction of <35% is an independent risk factor for mortality[22]. Despite this being true overall, prognoses within the above LVEF categories can vary widely, providing an opportunity for AI to categorise further into more clinically relevant sub-groups, possibly with the aid of novel AI derived imaging biomarkers.

Wu et al[23] utilised the power of natural language processing to review electronic hospital records and demonstrate that a large previously undiagnosed population fulfilled ESC criteria for HFpEF[24]. The same research group analysed these previously undiagnosed patients, in addition to confirmed HFpEF patients from the same hospital[25], according to self-defined ethnicity. They were able to demonstrate that white patients were older, with more atrial fibrillation, and had a worse 10 year survival from the first mention of heart failure in their notes, whereas black patients were younger with a more metabolic phenotype (hypertension, type 2 diabetes mellitus, obesity). This shows the potential of AI to acquire and then assess for clinically useful patterns; such defined sub-groups opens up the possibility of tailored treatments to improve outcomes.

Work by Gu et al in 2017 demonstrated a theoretical role for the importance of first-phase ejection fraction (EF1) in the assessment of cardiac function[26]. Its role was particularly prominent in the assessment of heart failure patients, predicting response to cardiac resynchronisation therapy in HFrEF[27] and in patients with severe aortic stenosis, who can develop heart failure due to raised afterload[28]. Ongoing work will utilise AI to incorporate EF1 measurement into an established auto-reporting software, and analyse the cohorts derived by Wu et al[23] according to the EF1 to assess its prognostic impact in HFpEF.

Beyond diagnosis, natural language processing and large language models (See Table 2) have a wide range of applications in heart failure, including improvement in assessment of medication adherence[29], prediction of readmission[30], assessment of care quality measures[31] and effectiveness of management strategies[32].

Due to the paucity of life-prolonging treatment in HFpEF, it is a particularly fertile ground for AI to sub-stratify patients into clusters who might respond to particular treatments. For example, Shah et al[33] used Support Vector Machines to analyse a wide variety of clinical data and identify separation boundaries between classes of interest in a much higher dimensional feature space[34]. Such clusters derived include natriuretic peptide deficiency syndrome, extreme cardiometabolic syndrome, and right ventricle-cardio-abdomino-renal syndrome.

## 4 Treatment

AI has advanced pharmacological therapies in several ways, including drug development, drug repurposing and tailoring treatment to specific patients or groups. Recent advances in AI have been used to guide pharmacological treatment strategies through analysis of Electronic Health Records, genomic data and trial results.

Machine Learning (ML) models analyse data and forecast which patients are likely to benefit from particular therapies. Amin et al[35] devised an AI model to predict the best type of medical management for individual patients with heart failure. The best combination of medication for each patient was decided based on risk stratification. They concluded that patients treated with the AI model-based recommendations led to a reduced rate of hospitalisation and death compared to the control population.

Halasz et al[36] showed that AI has a significant role to play in the field of early prediction of cardiac dysfunction related to cancer therapy, specifically anthracyclines. Cardiac dysfunction secondary to cardiotoxicity, leading to heart failure, is a significant risk of using anthracyclines and early detection of dysfunction is necessary to tailor cancer therapies accordingly. They proposed diagnosing LVSD using AI-based ECG algorithms instead of the reference method, echocardiography. They concluded this technique as cost-effective and faster and produced similar results to echocardiography in the diagnosis of LVSD. This may be beneficial as less specialised professionals could aid in faster diagnosis and earlier alteration of treatment leading to better overall outcomes.

AlphaFold is a deep learning-based model which predicts protein structures[37]. In cardiovascular medicine many conditions are linked to dysfunctional proteins, so identifying and targeting these proteins is key to developing new therapeutics for heart failure. Desai et al[38] discuss how the latest iteration of the model, AlphaFold 3, is able to streamline processes for drug design by predicting binding sites and optimal shapes for potential drug molecules. Desai et al describe how AlphaFold 3 analyses current data to identify novel applications for existing pharmacotherapy, potentially saving time and resources. By designing and tailoring drugs based on individual protein structures AlphaFold 3 can customise treatments based on genetic profiles.

Yang et al[39] devised a deep learning model to identify cardioprotective targets useful for treatment of dilated cardiomyopathy (DCM). They identified histone deacetylase 6 as a potential therapeutic target. They found that inhibition of this reduced sarcomere damage in mouse myocytes and protected against heart failure. They developed an inhibitor which had these effects which is promising for future therapeutic drugs to prevent or slow damage to cardiomyocytes in inherited DCM.

Pavlov et al[40] used a machine learning algorithm to assess the impact of current cardiovascular medication on patient outcomes. Their research suggested that initiation of beta blockers and sacubitril/valsartan therapy at the same time as SGLT2 inhibitors produced the most favourable outcomes.

Bucker et al’s study on AI-guided decisions on pharmacotherapy[41] concluded that, while the area showed promise, there was still significant work to be done in this field. The model they developed did not include patient preference or shared decision making. They did suggest future AI-based discussions as a possible preference for patients due to time constraints. Bucker et al[41] felt that the current models require a certain level of expert human oversight to guide decision-making.

## 5 Conclusion

Our review describes the many uses of AI in the heart failure sphere, and the manner in which they have already improved efficiency and diagnostic ease through a wide variety of methods. The clear benefits of applying AI to heart failure must be shared with partners in the developing world, to ensure that the maximum benefit to the patient can be gained from medical environments with more limited resources. As earlier, more accurate AI-aided diagnoses can be made, and modern, AI-derived, personalised, life-prolonging drugs can be provided to patients, the authors are hopeful that in twenty years’ time, AI will have significantly enhanced the way the field of heart failure cardiology is practised worldwide.

## 6 Future Perspectives

The importance of AI in a disease such as heart failure has the capacity to revolutionise the way in which cardiologists and generalists approach the disease over the next twenty years.

However, medicine must not be blind to disparities in healthcare provision, and cardiovascular medicine is no different. Inequity is based on a large number of factors with complex relationships to each other, including socioeconomic, ethnicity and gender, among others. As we know these disparities exist, we must take every effort to ensure that AI training datasets account for these disparities, to minimise bias in AI models and subsequent advances in disease prevention, and improvements in morbidity and mortality.

If these disparities are not taken into account, then bias will be perpetuated and possibly exaggerated; to perpetuate such inequality in the face of AI’s ongoing development and expansion would represent a grave abrogation of responsibility which the authors would consider highly irresponsible.

As such, a key role of AI in heart failure should be the reassessment of existing paradigms (for example, diagnostic scoring systems and prognosis) based on data from racially and socially diverse populations, who will have varied risk profiles based on their genetics and co-morbidities.

The authors believe the greatest advances in the coming years will be in the utility of AI to enable diagnosis in low resource settings (with cheap equipment, by lower skilled clinicians), and as such enable prompt and effective commencement of evidence-based treatment; these are the settings where the greatest gains stand to be made, and where appropriate resources should be channelled if possible.

## Figures and Tables

**Table 1:** A glossary of commonly used AI terms

Keyword	Explanation
Artificial Intelligence	A broad term that describes the novel ability of computer based systems to mimic human cognitive function. These systems can assess a wide variety of data for patterns and solve complex problems.
Convolutional Neural Network	A system that learns features by itself via multiple filtering layers. Particularly useful in image and video analysis.
Deep Learning	The use of multiple layered neural networks to analyse large data sets. Common applications include voice recognition, whereby the system can analyse complex data without supervision.
Deep Neural Network	A neural network with many (hidden) layers between input and output. Aims to mimic the human brain in processing large amounts of input data.
Machine Learning	A field within Artificial Intelligence that is focussed on the derivation of algorithms from input data, which can then be used to make predictions. Performance improves with exposure to more data.
Natural Language Processing	A field within Artificial Intelligence that describes the processing of the gamut of data within human language and the formation of meaningful responses.
Support Vector Machine	A set of supervised learning methods for classification, regression, and outlier detection tasks.

**Table 2:** A glossary of novel concepts in Large Language Models

Keyword	Explanation
Autoregressive Models	Text generation based on the word(s) that came directly before (eg. Chat GPT)
Embeddings	Encoding of semantic relationships (eg opposites, similes)
Large Language Model	An AI technology that can understand and generate human language text, trained on large amounts of data, and based on deep learning architecture such as transformers.
Masked Language Modelling	A large language model technique where the system is is trained on text with missing words. This improves question answering.
Tokenisation	Separation of text into smaller units, allowing the Large Language Model to analyse more effectively.
Top-k sampling	When generating text, this restricts word selection to a set number of probable words.
Transformer XL	Allows models to read large chunks of text without losing over-arching context.

## Plain Language Summary

Heart failure is common. It leads to periods of being unwell and death. Details about these patients are kept by hospitals. Artificial Intelligence (AI) can analyse these, to work out how likely heart failure is. AI can also suggest how likely it is that these patients will become more unwell. AI can help with initial diagnosis, and tell you whether certain drugs will work. This is true for different types of heart failure, including “Heart Failure with Preserved Ejection fraction”, which is often overlooked as a diagnosis. We believe that AI will improve the quality and length of life in people with heart failure.

## Article Highlights

- Heart failure is a disease with significant morbidity and mortality; healthcare systems store a large amount of data regarding patients with this condition.
- Artificial Intelligence allows analysis of large quantities of this data and prediction of trends that would be difficult and time consuming for humans to achieve.
- ECGs are cheap to perform, and a fertile ground for AI to predict diagnosis of heart failure
- AI can help guide and report echos, allowing less-skilled professionals to perform them, potentially improving availability in resource poor settings.
- AI allows categorisation within the HF population into sub-sets based on ejection fraction (HFrEF v HFpEF) and potential respondse to treatment.
- AI can predict cardiotoxic responses of chemotherapy
- AI can suggest new drug targets for heart failure.
- As AI expands in heart failure, care must be taken to prevent bias in the training data sets.
- AI should allow for more equitable healthcare provision in heart failure, and not entrench existing disparities between communities and countries.
